# Role of PII proteins in nitrogen fixation control of *Herbaspirillum seropedicae *strain SmR1

**DOI:** 10.1186/1471-2180-11-8

**Published:** 2011-01-11

**Authors:** Lilian Noindorf, Ana C Bonatto, Rose A Monteiro, Emanuel M Souza, Liu U Rigo, Fabio O Pedrosa, Maria BR Steffens, Leda S Chubatsu

**Affiliations:** 1 National Institute of Science and Technology for Biological Nitrogen Fixation, Department of Biochemistry and Molecular Biology, Universidade Federal do Paraná, CP 19046, Curitiba, PR, 81531-980, Brazil; 2 Department of Genetics, Universidade Federal do Paraná, CP 19071, Curitiba, PR, 81531-980, Brazil

## Abstract

**Background:**

The PII protein family comprises homotrimeric proteins which act as transducers of the cellular nitrogen and carbon status in prokaryotes and plants. In *Herbaspirillum seropedicae*, two PII-like proteins (GlnB and GlnK), encoded by the genes *glnB *and *glnK*, were identified. The *glnB *gene is monocistronic and its expression is constitutive, while *glnK *is located in the *nlmAglnKamtB *operon and is expressed under nitrogen-limiting conditions.

**Results:**

In order to determine the involvement of the *H. seropedicae glnB *and *glnK *gene products in nitrogen fixation, a series of mutant strains were constructed and characterized. The *glnK^- ^*mutants were deficient in nitrogen fixation and they were complemented by plasmids expressing the GlnK protein or an N-truncated form of NifA. The nitrogenase post-translational control by ammonium was studied and the results showed that the *glnK *mutant is partially defective in nitrogenase inactivation upon addition of ammonium while the *glnB *mutant has a wild-type phenotype.

**Conclusions:**

Our results indicate that GlnK is mainly responsible for NifA activity regulation and ammonium-dependent post-translational regulation of nitrogenase in *H. seropedicae*.

## Background

The PII family comprises homotrimeric proteins that have important roles in the control of the central metabolism in bacteria and plants, acting as transducers of the cellular nitrogen and carbon levels [1,2]. In many *Proteobacteria *studied there is a pair of PII proteins, usually called GlnB and GlnK, and their function is to sense the cellular levels of nitrogen, carbon and energy by binding the effectors 2-oxoglutarate, ATP and ADP [2,3]. These signals are then relayed to target proteins through conformational changes triggered by interaction with the effectors. The proteobacterial PII proteins also undergo a cycle of uridylylation/deuridylylation catalyzed by the bifunctional GlnD protein [1] in response to the intracellular levels of nitrogen. These conformational and covalent state changes stimulate or inhibit interactions of PII with different cellular protein targets involved in nitrogen and carbon metabolism [2].

PII proteins are key players in the regulation of nitrogen fixation in *Proteobacteria*. In *Klebsiella pneumoniae *and *Azotobacter vinelandii*, GlnK is required to regulate the activity of NifL, which inhibits NifA, the *nif *gene specific activator, under nitrogen-excess conditions [4-6]. In *Azospirillum brasilense *and *Rhodospirillum rubrum *GlnB is necessary for the activation of NifA under nitrogen-limiting conditions [7-9], whereas in *Rhodobacter capsulatus *both PII proteins are necessary for the NH_4_^+^-dependent regulation of NifA activity [10]. In addition, PII proteins are also involved in the post-translational control of nitrogenase activity in *R. rubrum *[11] and in *A. brasilense *through interaction with DraT, DraG and AmtB [12].

*Herbaspirillum seropedicae *is a nitrogen-fixing *β-Proteobacterium *isolated from the rhizosphere and tissues of several plants, including economically important species [13]. In this organism two PII-like coding genes were identified, *glnB *and *glnK *[14,15]. The *glnB *gene is monocistronic and its expression is constitutive [14], whereas *glnK *is apparently co-transcribed with *amtB *and *orf1*, which encode for an ammonium transporter and a membrane associated protein of unknown function, respectively [15]. Recently *orf1 *was named *nlmA *(*n*itrogen *l*imitation *m*embrane protein A) since its product was detected in membrane extracts of *H. seropedicae *grown under nitrogen-limitation conditions [16]. The expression of the *nlmAglnKamtB *operon is dramatically increased under nitrogen-limiting conditions and is dependent on NtrC [15]. As in other *Proteobacteria*, both PII proteins from *H. seropedicae *are targets of covalent modification by GlnD (uridylyl-transferase/uridylyl removing enzyme) in response to the levels of ammonium ions [17].

## Results and Discussion

To analyze the role of GlnK and GlnB in the control of nitrogen fixation in *H. seropedicae*, *glnB *(LNglnB) and *glnK *(LNglnK) insertional mutants and a *glnK *in-frame deletion mutant strain (LNglnKdel) were constructed and their phenotypes analyzed under different physiological conditions. These mutant strains were able to grow using nitrate as sole nitrogen source (data not shown).

The effect of *glnB *and *glnK *disruption on the NtrC-dependent expression of the *nlmAglnKamtB *operon [15] was determined using chromosomal *amtB*::*lacZ *transcriptional fusions of strains LNamtBlacZ, LNglnBamtBlacZ and LNglnKamtBlacZ. These strains were grown under N-limiting (5 mmol/L glutamate or 2 mmol/L NH_4_Cl) or N-excess (20 mmol/L NH_4_Cl) conditions and assayed for β-galactosidase. The LNamtBlacZ strain grown under N-limiting conditions showed β-galactosidase activity 21 times higher than in high ammonium (Table 1), confirming that *nlmAglnKamtB *is highly expressed under N-limiting conditions [15]. Strains LNglnKamtBlacZ and LNglnBamtBlacZ revealed a similar pattern of *amtB *expression, indicating that the mutation of either *glnK *or *glnB *does not affect *nlmAglnKamtB *expression. Since *nlmAglnKamtB *transcription is NtrC-dependent, these results suggest that GlnB and GlnK can substitute for each other in control of the NtrC/NtrB system in *H. seropedicae*. In agreement with this suggestion, *ntrC *[18] and *glnD *(unpublished results) mutants strains of *H. seropedicae *are unable to grow on nitrate, whereas the *glnB *and *glnK *mutant strains can use nitrate as sole nitrogen source.

**Table 1:** Effect of *glnB *and *glnK *mutations on *nlmAglnKamtB *expression

Growth Conditions	β-galactosidase Activity [nmol *o*-nitrophenol/(min.mg protein)]		
	
	Strains		
	
	LNamtBlacZ (SmR1, *amtB::lacZ*)	LNglnKamtBlacZ (Δ*glnK*, *amtB::lacZ*)	LNglnBamtBlacZ (*glnB*-Tc^R^, *amtB::lacZ*)
5 mmol/L glutamate	(2.5 ± 0.2) × 10^3^	(2.4 ± 0.2) × 10^3^	(2.3 ± 0.2) × 10^3^

2 mmol/L NH_4_Cl	(2.1 ± 0.1) × 10^3^	(2.29 ± 0.08) × 10^3^	(2.2 ± 0.1) × 10^3^

20 mmol/L NH_4_Cl	(1.1 ± 0.2) × 10^2^	(1.4 ± 0.4) × 10^2^	(1.6 ± 0.3) × 10^2^

Indicated strains of *H. seropedicae *were grown in the presence of glutamate or NH_4_Cl. β-galactosidase activity was determined as described. Values are the mean of at least three independent experiments ± standard deviation.

In *Escherichia coli *both GlnB and GlnK are involved in the regulation of NtrC phosphorylation by NtrB, although GlnB is more effective [19]. Although several attempts were made, we failed to construct a double *glnBglnK *mutant suggesting that an essential role is shared by these proteins in *H. seropedicae*.

**Figure 1:**
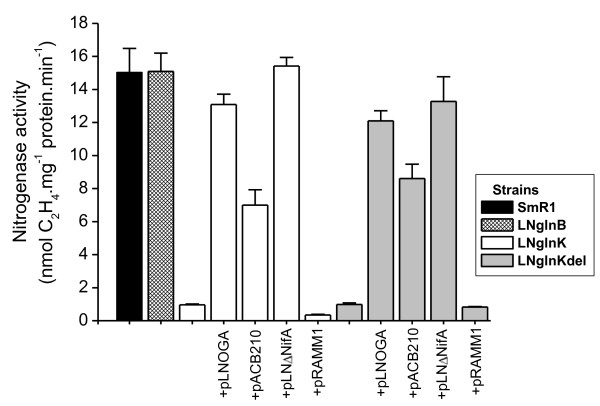
**Nitrogenase activity of *H. seropedicae *wild-type, *glnB *and *glnK *strains**. Nitrogenase activity was determined as described using strains SmR1 (wild-type), LNglnB (*glnB*-Tc^R^), LNglnK (*glnK*-Km^R^), LNglnKdel (Δ*glnK*) grown in semi-solid medium. The *glnK *mutants carrying plasmids pLNOGA, pACB210, pLNΔNifA or pRAMM1, which respectively express NmlA-GlnK-AmtB, GlnB, ΔN-NifA and NifA were also evaluated. Data represent the average of at least three independent experiments and bars indicate the standard deviations.

The effect of *glnK *or *glnB *mutation on nitrogenase activity of *H. seropedicae *was determined in cultures grown in NH_4_^+^-free semi-solid NFbHP medium (Figure 1). Nitrogenase activity was reduced by approximately 95% in both *glnK *strains (LNglnKdel and LNglnK) indicating that GlnK is required for nitrogenase activity in *H. seropedicae*. On the other hand, the *glnB *strain (LNglnB) showed activity similar to that of the wild-type. These results contrast with those reported by Benelli et al [14] who constructed a *H. seropedicae glnB*::Tn5*-20B *mutant (strain B12-27) that was unable to fix nitrogen. Immunoblot assays did not detect GlnK in the B12-27 strain [Additional file 1: Supplemental Figure S1], suggesting that a secondary recombination event may have happened in this strain resulting in loss of GlnK not observed by Benelli et al [14].

The nitrogenase phenotype of the *glnK *mutants was complemented by pLNOGA (*nlmAglnKamtB*) and also partially restored (about 50%) by a plasmid expressing *glnB *under control of its own promoter (pACB210) suggesting that a higher copy number of *glnB *can substitute for *glnK *under N-limitation. The lower nitrogenase activity of the *glnK *strains could be due to lack of *nif *expression or inhibition of nitrogenase. We therefore analyzed the effect of the *glnK *mutation on the NtrC-dependent *nifA *promoter [20] and on the NifA-dependent *nifB *promoter of *H. seropedicae *[21] by using plasmids carrying *nifA::lacZ *(pRW1) or *nifB::lacZ *(pEMS140) fusions (Table 2). The β-galactosidase activity was the same in both wild-type (SmR1) and *glnK *(LNglnK) strains containing *nifA::lacZ*, supporting the view that GlnK is not strictly necessary for NtrC regulation in *H. seropedicae *in the presence of a functional *glnB *gene. On the other hand, expression of the *nifB::lacZ *fusion was reduced 10-fold in the *glnK *mutant compared to the wild-type, indicating that GlnK is required for *nifB *expression in *H. seropedicae*, even in the presence of wild type *glnB*. These results indicate that the lower nitrogenase activity in the *glnK *mutants was the result of lack of *nif *expression, most likely due to impaired NifA activity.

**Table 2:** Promoter activity of *nifA*::*lacZ *and *nifB*::*lacZ *fusions in *H. seropedicae *wild-type (SmR1) and *glnK *mutant (LNglnK) strains

Strains	β-galactosidase Activity [nmol *o*-nitrophenol/(min.mg protein)]			
	
	Plasmids			
	
	none	pPW452 (promoter-less *lacZ *vector)	pRW1 (*nifA*::*lacZ*)	pEMS140 (*nifB*::*lacZ*)
SmR1	(3 ± 1) × 10	(6 ± 2) × 10	(7 ± 1) × 10^2^	(2.8 ± 0.1) × 10^3^

LNglnK	(2.0 ± 0.7) × 10	(4 ± 2) × 10	(6 ± 1) × 10^2^	(2.5 ± 0.3) × 10^2^

*H. seropedicae *strains carrying the indicated plasmids were grown in NFbHP medium supplemented with 10 mmol/L of NH_4_Cl under air at 30°C. The cells were then centrifuged, resuspended in NFbHP (nitrogen-free) medium and de-repressed for 7 hours under 1.5% oxygen. β-galactosidase was determined as described. Values are averages of at least three independent experiments ± standard deviation

Previous results showed that the N-terminal domain of *H. seropedicae *NifA is required for controlling its activity in response to NH_4_^+^, and that an N-truncated form of NifA is transcriptionally active, but not responsive to NH_4_^+ ^levels [22,23]. Thus, the nitrogenase activity was determined in the *glnK *mutants carrying pRAMM1 or pLNΔNifA which express a full NifA and an N-truncated form of NifA, respectively (Figure 1). The nitrogenase activity of the *glnK *mutants was restored only by the N-truncated-NifA protein, reinforcing the indication that the nitrogenase negative phenotype of *glnK *strain is due to the presence of an inactive NifA.

**Figure 2:**
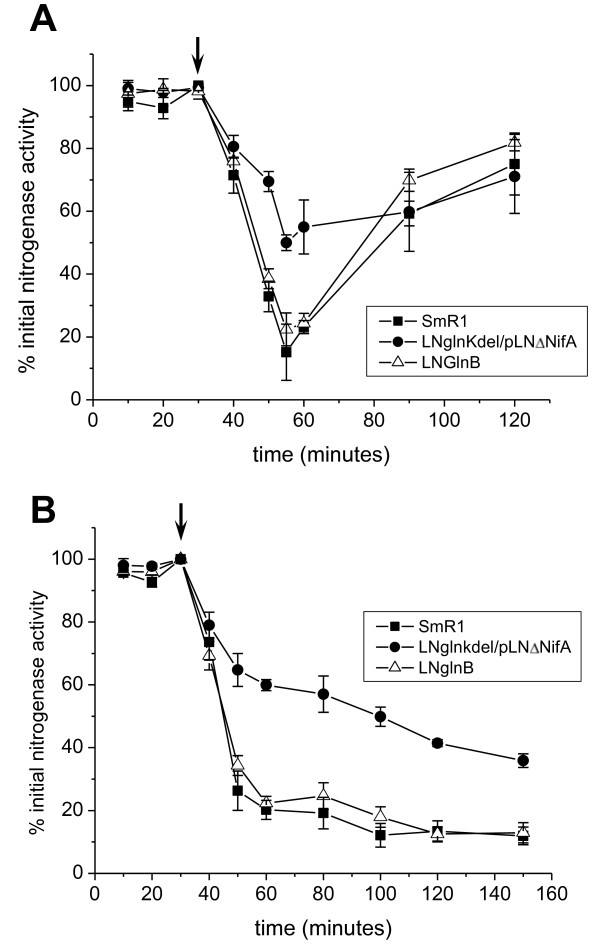
**Effect of ammonium ions on nitrogenase activity in *H. seropedicae *wild-type, *glnB *and *glnK *strains**. Nitrogenase switch-off/on of *H. seropedicae *wild-type, *glnB *and *glnK *carrying plasmid pLNΔNifA was performed as described. Cells were grown under nitrogenase de-repressing conditions when NH_4_Cl was added (arrow). Samples were analyzed 10, 20 and 30 minutes after acetylene injection to confirm linear nitrogenase activity. **Panel A**: addition of NH_4_Cl (0.3 mmol.L^-1^). **Panel B**: addition of NH_4_Cl (1 mmol.L^-1^). The results represent the average of experiments with three independent cultures and bars indicate the standard deviation.

Nitrogenase activity is reversibly inhibited by addition of ammonium or energy depletion in several diazotrophs, a phenomenon called nitrogenase switch-off. The best studied process is the reversible NifH ADP-ribosylation carried out by the DraT and DraG enzymes whose activities are controlled by processes involving PII proteins at least in some diazotrophs [11,12,24,25]. A PII protein has also been implicated in the control of nitrogenase by direct interaction with NifH in the methanogenic archaeon *Methanococcus maripaludis *[26]. In *H. seropedicae *reversible ADP-ribosylation of NifH by the DraT/DraG does not occur since *draTG *genes are absent [27] [GenBank:CP002039]. Although the mechanism of NH_4_^+^-dependent nitrogenase control in this organism is not known, it is thought to be due to change in prevailing physiological conditions leading to nitrogenase inhibition. Since the *glnK *mutant is Nif^-^, we used strain LNglnKdel carrying plasmid pLNΔNifA for the switch-off experiments. Addition of low concentrations of NH_4_Cl (300 μmol/L) to derepressed cells caused an inactivation of nitrogenase (Figure 2A). Wild-type and *glnB *strains retained less than 20% of initial nitrogenase activity 25 minutes after ammonium addition, which was restored to 60-70% of initial activity 60 minutes after ammonium addition. This effect does not involve protein synthesis since the presence of chloramphenicol or tetracycline had no effect on this behavior [28]. Although nitrogenase of strain LNglnKdel/pLNΔNifA was partially inhibited by ammonium addition, the strain retained about 50% of its initial activity, indicating only a partial nitrogenase switch-off (Figure 2A). After addition of 1 mmol/L of NH_4_Cl (Figure 2B) the activity of the wild-type and *glnB *strains dropped sharply to less than 30% and did not recover even 120 minutes after ammonium addition. In contrast, 40% of the initial nitrogenase activity was still present in the *glnK *strain 120 minutes after ammonium addition and the decrease in nitrogenase activity was slower: 20 minutes after ammonium addition the wild-type had only 25% activity, whereas the *glnK *strain had about 65% of the original nitrogenase activity. These results indicate that GlnK is involved in the nitrogenase inactivation by NH_4_^+ ^in *H. seropedicae*, and that GlnB cannot fully replace GlnK in triggering nitrogenase switch-off. It is interesting to note that there was also a delay in nitrogenase reactivation in the *glnK *mutant (Figure 2A), which may suggest that GlnK is involved in both nitrogenase inactivation by NH_4_^+ ^and re-activation upon NH_4_^+ ^depletion.

Recently results using a proteomic approach [16] showed that *H. seropedicae *GlnK is associated with the membrane at higher concentration immediately after addition of ammonium. This membrane association was shown to be AmtB-dependent, as shown in several bacteria [2,16]. Previous results also showed that an *amtB *mutant has a partial NH_4_^+ ^switch off very similar to that shown by the *glnK *mutant[15]. These results allow us to propose a model for the regulation of nitrogen fixation in *H. seropedicae*. Under N-limiting conditions, NtrC-dependent promoters are activated leading to expression of *nifA *and *nlmAglnKamtB *genes. The status of fixed nitrogen is signaled to NtrC via the uridylylation state of either GlnB or GlnK. Under a low ammonium and oxygen condition, NifA activates the expression of *nif *genes in a process which requires GlnK, most probably in an uridylylated form. Thus, under N-limiting conditions the nitrogenase complex is active, AmtB is associated with the membrane, NlmA is most probably in the periplasm and GlnK is mainly located in the cytoplasm. When ammonium is added, deuridylylated GlnK rapidly associates with the cell membrane by interacting with AmtB to form the GlnK-AmtB complex which, in turn, signals to nitrogenase to switch-off by a yet unknown process.

## Conclusions

In summary, our results show that both GlnB and GlnK proteins can regulate NtrC-dependent promoters in *H. seropedicae*. Under physiological conditions, GlnK is required for NifA activity control. GlnK also controls the nitrogenase switch-off in response to NH_4_^+ ^by a mechanism which most probably involves the formation of a membrane-bound GlnK-AmtB complex.

## Methods

### Plasmids, Bacterial strains and Growth conditions

The *H. seropedicae *and *E. coli *strains and plasmids used in this work are listed in Table 3. *E. coli *strains were grown routinely in Luria medium (Luria broth or Luria agar) [29] at 37°C. *H. seropedicae *was grown at 30°C in NFbHP medium [30] supplemented with NH_4_Cl (20 mmol/L) or the indicated nitrogen source. The concentrations of the antibiotics used were as follows: ampicillin (250 μg/mL), tetracycline (10 μg/mL), kanamycin (100 μg/mL for *E. coli*, 1 mg/mL for *H. seropedicae*), streptomycin (80 μg/mL) and choramphenicol (30 μg/mL for *E. coli*, 100 μg/mL for *H. seropedicae*).

**Table 3:** *Herbaspirillum seropedicae *strains and plasmids

Strains	Phenotype/genotype	Reference
*Herbaspirillum seropedicae*		
SmR1	Wild type, Nif^+^, Sm^R^	[38]
LNglnK	SmR1 containing *glnK::sacB*- Km^R^	this work
LNglnKdel	SmR1 containing Δ*glnK*	this work
LNglnB	SmR1 containing *glnB*::Tc^R^	this work
LNamtBlacZ	SmR1 containing a*mtB*::*lacZ*-Km^R^	this work
LNglnKamtBlacZ	LNglnKdel containing a*mtB*::*lacZ*-Km^R^	this work
LNglnBamtBlacZ	LNglnB containing a*mtB*::*lacZ*-Km^R^	this work
B12-27	SmR1 containing *glnB::*Tn5-*20B*	[14]
*Escherichia coli*		
DH10B	Sm^r^; F^' ^[*proAB*^+ ^*lacZ*ΔM15]	Life Technologies
S17.1	Sm^R^, Tra^+ ^*pro thi recA hsdR *(RP4-2 *kan*::Tn7 *tet*::Mu)	[39]
**Plasmids**	**Relevant characteristics**	**Reference**
pACB192	1.7 kb DNA fragment containing the *glnB *gene of *H. seropedicae *in pSUP202	This work
pACB194	*glnB *gene of *H. seropedicae *with a tetracycline resistance transposon EZ::TN™ < TET-1 > (Epicentre) in pSUP202	this work
pACB210	*glnB *gene of *H. seropedicae *in pLAFR3.18Cm	this work
pDK6	Expression vector/*tac *promoter, Km^R^	[37]
pDK6nifACT	*H. seropedicae nifA *deleted of 606 bp in the 5'coding region cloned into pDK6 carrying the *nifA *promoter	this work
pDK6pnifA	*nifA *gene promoter region of *H. seropedicae *in pDK6	this work
pEMS140	*nifB*-*lacZ *transcriptional fusion *of H. seropedicae *in pPW452	[21]
pEMS301	1.7 kb *Eco*RI fragment that contains the promoter region and part of the *nifA *gene of *H. seropedicae *in pTZ19R	[40]
pLAFR3.18Cm	Tc^R^, Cm^R^, IncP cosmid with the pTZ18R cloning nest	[15]
pLNΔNifA	Expresses ΔN-NifA of *H. seropedicae *with its own promoter in pLAFR3.18Cm	this work
pLNOGA	5.1 kb fragment that contains the *nlmAglnKamtB *operon of *H. seropedicae *in pLAFR3.18Cm (former named pLARF3.18OGA)	[15]
pLNglnK	0.9 kb *Bam*HI/*Hin*dIII fragment that contains the 3' terminal of the *nlmA *gene, the complete *glnK *gene and 5' terminal of the *amtB *gene of *H. seropedicae *in pTZ18R	this work
pMH1701	Km^R^, contains a *sacB*-Km^R ^cassette	[35]
pPW452	Tc^R^, transcriptional *lacZ *gene fusion	[41]
pRAM2T7	contains *H. seropedicae nifA *deleted of 606 bp in the 5'end, encoding an N-truncated form of NifA deleted of its N-terminal domain and Q-linker	this work
pRAMM1	*nifA *of *H. seropedicae *in pLAFR3.18Cm	this work
pRW1	*nifA*-*lacZ *transcriptional fusion *of H. seropedicae *in pPW452	[20]
pSUP202	Ap^R^, Cm^R^, Tc^R^, Mob	[39]
pSUPamtBClacZ	Central region of the *amtB *gene with a *lacZ*-Km^R ^cassette insertion in pSUP202	[15]
pSUPglnK	0.9 kb *Bam*HI/*Hin*dIII fragment that contains the 3' terminal of the *nlmA *gene, the complete *glnK *gene and 5' terminal of the *amtB *gene of *H. seropedicae *in pSUP202	this work
pSUPglnKdel	Δ*glnK *(192bp) gene of *H. seropedicae *in pSUP202	this work
pSUPglnKdelsacB	contains Δ*glnK *and a *sacB*-Km^R ^cassette (from pMH1701) cloned into the vector pSUP202	this work
pSUPglnKsacB	0.9 kb fragment spanning from the 3'end of *nlmA *to the 5'end of *amtB *with a *sacB*-Km^R ^(from pMH1701) inserted into the *glnK *gene	this work
pTZ19R	Ap^R ^*lacZ f*1 IG	[42]
pUC18	Ap^R^, *lacZ*, *f1*	Invitrogen
pUCG08del	0.8 kb DNA fragment that contains the 3' terminal of the *nlmA *gene, the complete *glnK *gene and the 5' terminal of the *amtB *gene of *H. seropedicae *in pUC18.	this work
pUCglnKdel	Δ*glnK *gene of *H. seropedicae *in pUC18	this work

### Enzyme assays

β-galactosidase activity was determined in cells carrying a *lacZ *fusion as described [31]. To study the *amtB*-*lacZ-*Km^R ^chromosomal fusion expression, *H. seropedicae *strains carrying chromosomal transcriptional fusions were grown for 14 hours in NFbHP medium containing glutamate (5 mmol/L) or NH_4_Cl (2 mmol/L or 20 mmol/L), and assayed for β-galactosidase activity. To study the *nifA *and *nifB *expression, *H. seropedicae *strains carrying plasmid-borne transcriptional fusions *nifA*::*lacZ *or *nifB*::*lacZ *were grown for 14 hours in NFbHP medium containing NH_4_Cl (10 mmol/L) under air at 30°C. The cells were then centrifuged, resuspended in 3 mL of NFbHP medium (O.D._600 _= 0.2) and incubated in 25 mL flasks, at 30°C for 7 hours under 1.5% oxygen. The results are reported as nmol of *o*-nitrophenol (NP) produced per min per mg protein. Protein concentration was determined by the Bradford method [32] using bovine serum albumin as standard.

Nitrogenase activity was determined using cells grown in semi-solid NFbHP medium containing glutamate (0.5 mmol/L). For nitrogenase switch-off/on assays cells were grown in liquid NFbHP medium with glutamate (4 mmol/L) at 30°C and 120 rpm [28]. Nitrogenase activity was determined by acetylene reduction [33,34].

### Construction of the LNglnB mutant of *H. seropedicae*

Plasmid HS26-FP-00-000-021-E03 (Genopar consortium, http://www.genopar.org), which contains the *H. seropedicae glnB *gene in pUC18, was linearized with *Eco*RI and treated with T4DNA polymerase. It was then digested with *Hin*dIII to release a 1.7 kb fragment containing the *glnB *gene. This fragment was subcloned into the vector pSUP202 previously linearized with *Bam*HI, treated with T4DNA polymerase and digested with *Hin*dIII to produce plasmid pACB192.

*In vitro *transposon mutagenesis of the *glnB *gene carried by plasmid pACB192 was performed using the *EZ::TN*™ <*TET-1*> Insertion Kit (Epicentre Technologies) following the manufacturer's instructions. A plasmid containing the transposon insertion in the *glnB *coding region was selected and named pACB194. This plasmid was introduced by conjugation to *H. seropedicae *SmR1 using *E. coli *strain S17.1 as the donor. Recombinant colonies were selected for tetracycline resistance and screened for the loss of chloramphenicol resistance (vector marker). Southern blot of restriction enzyme digested genomic DNA was used to confirm the presence of the transposon in the *glnB *gene (data not shown). This *H. seropedicae glnB-*Tc^R ^strain was named LNglnB.

### Construction of the LNglnK mutant of *H. seropedicae*

To clone the *glnK *gene, chromosomal DNA of *H. seropedicae *was amplified using the primers glnKD (5'-GACTGAAAGGATCCGCGTGTCC-3', *Bam*HI restriction site is underlined) and glnKR (5'-CGAGGGCAAAGCTTCTTCGGTGG-3', *Hind*III restriction site is underlined). The amplified fragment was then ligated into *Bam*HI/*Hind*III-cut pTZ18R, generating the plasmid pLNglnK. This BamHI/HindIII fragment containing the *glnK *gene was then subcloned into pSUP202, yielding plasmid pSUPglnK. A *sacB*-Km^R ^cassette excised with *Bam*HI from pMH1701 [35] was inserted into the *Bgl*II site of the *glnK *gene. The resulting plasmid (pSUPglnKsacB) was transferred into *H. seropedicae *SmR1 by conjugation using *E. coli *strain S17.1 as the donor. Mutant colonies were selected for kanamycin resistance and screened for the loss of chloramphenicol resistance, as before. Hybridization of genomic DNA was used to confirm the presence of the cassette in the *glnK *gene (data not shown). This *glnK-*Km^R ^mutant was named LNglnK.

### Construction of the LNglnKdel mutant of *H. seropedicae*

To construct a mutant containing an in-frame 192 bp deletion of the *glnK *gene, plasmid pUCG08del containing the 3' terminus of the *nlmA *gene, the *glnK *gene and the 5' terminus of the *amtB *gene was used as a template in two distinct PCR reactions. Primers M13universal and GlnKdelR (5' AAGCCCTCGAGTTCAGTCACGGT 3', *Xho*I restriction site is underlined) were used to amplify a 180 bp region upstream of *glnK *and the first 107 bp of the *glnK *gene. The primers M13reverse and GlnKdelD (5' GGACCTGCTCGAGGTGATCCGT 3', *Xho*I restriction site is underlined) were used to amplify the last 58 bp of the *glnK *gene and the first 180 bp of *amtB*. The amplified fragments were joined by the *Xho*I sites. This fragment containing *glnK *deleted of 192 bp was then used as template for a PCR reaction with the primers M13reverse and M13universal. The resulting PCR product was digested with *Bam*HI and *Pst*I and inserted into pUC18 to give pUCglnKdel. This fragment was then subcloned into pSUP202, yielding the plasmid pSUPglnKdel. A *sacB*-Km^R ^cassette excised with *Bam*HI from pMH1701 [35] was inserted into the vector region of the *Bam*HI-cut pSUPglnKdel plasmid. The resulting plasmid (pSUPglnKdelsacB) was conjugated into *H. seropedicae *SmR1 using *E. coli *strain S17.1 as the donor. Recombinant colonies were selected for kanamycin and chloramphenicol resistance. One mutant strain was selected, and grown overnight in liquid NFbHP medium supplemented with ammonium chloride (20 mmol/L) and 80 μg/mL streptomycin. One microliter of the culture was plated on solid NFbHP medium supplemented with 20 mmol/L NH_4_Cl, 5% sucrose and 80 μg/mL streptomycin. Sucrose is toxic to bacteria containing the *sacB *gene in the chromosome, therefore only strains that lost the *sacB*-Km^R ^cassette by a second homologous recombination event would grow. The selected strains were analyzed by PCR with the primers GlnKF1 (5'TGTCCAAGACCTTCGACG3') and GlnKR1 (5'CATGCTCATTAGAGTTCC3') which were homologous to the *glnK *flanking 5'- and 3'- regions, confirming the deletion of the 192 bp *glnK *fragment (data not shown). This in-frame *glnK *strain (ΔglnK) was named LNglnKdel.

### Construction of plasmid pLNΔNifA

An Eco47III/SacI DNA fragment containing the *nifA *gene promoter region of *H. seropedicae *was excised from the plasmid pEMS301[36] and sub-cloned into the SmaI/SacI-cut vector pDK6 [37], yielding plasmid pDK6pnifA. An *Xba*I DNA fragment encoding for the central and C-terminal region of NifA protein (ΔN-NifA) of *H. seropedicae *was excised from the plasmid pRAM2T7 and sub-cloned into the XbaI-cut pDK6pnifA, in the same orientation as the *nifA *promoter, yielding plasmid pDK6nifACT. Finally, a SacI/HindIII DNA fragment containing the *nifA *5'-truncated gene was excised from pDK6nifACT and sub-cloned into pLAFR3.18Cm digested with *Sac*I and *Hin*dIII. The generated plasmid was named pLNΔNifA and encodes for the central and C-terminal domains of NifA under control of the *nifA *promoter.

### Construction of the plasmid pACB210

A 1.7 kb EcoRI-HindIII fragment containing the *glnB *gene with its promoter region was excised from the plasmid HS10-MP-00-000-014-E08 (Genopar consortium, http://www.genopar.org), and sub-cloned into the vector pLAFR3.18 digested with EcoRI-HindIII to yield plasmid pACB210.

### Construction of chromosomal *amtB::lacZ *transcriptional fusions

To construct *amtB*-*lacZ *transcriptional fusions, the suicide plasmid pSUPamtBClacZ was introduced by conjugation, using *E. coli *strain S17.1 as the donor, into *H. seropedicae *strains SmR1, LNglnKdel and LNglnB resulting in the strains LNamtBlacZ, LNglnKamtBlacZ and LNglnBamtBlacZ, respectively. Genomic DNA hybridization confirmed the presence of the cassette *lacZ-*Km^R ^in the *amtB *gene (data not shown).

## List of Abbreviations

Ap^R^: ampicillin resistance; Cm^R^: chloramphenicol resistance, Km^R^: kanamycin resistance; Tc^R^: tetracycline resistance; Sm^R^: streptomycin resistance.

## Authors' contributions

LN constructed plasmids and *H. seropedicae *mutants, carried out physiological experiments and helped to draft the manuscript; ACB constructed plasmids and carried out immunoassays; RAM constructed plasmids and designed some of the experiments; LN, RAM and LUR helped to draft the manuscript; FOP, EMS, MBRS and LSC conceived the study, participated in its design and in writing the manuscript, LSC also supervised the study. All authors read and approved the final manuscript.

## Supplementary Material

Additional file 1 **Immunoblot analysis of *H. seropedicae *PII proteins**. Click here for file
