# Supplementary material for: Role of PII proteins in nitrogen fixation control of Herbaspirillum seropedicae strain SmR1
Source: BMC Microbiol. 2011 Jan 11;11:8. doi: 10.1186/1471-2180-11-8 (PMC3023670; doi:10.1186/1471-2180-11-8)
Supplement: Additional file 1 — Immunoblot analysis of H. seropedicae PII proteins. [file 1471-2180-11-8-S1.DOC]

**Additional file**

**Immunoblot analysis of *H. seropedicae* PII proteins**

The nitrogenase activity of the *glnB* knockout mutant strain LNglnB was similar to that of the wild-type [Figure 1]. This result contrasts with that reported by Benelli et al [Supplemental Reference S1], who constructed an *H. seropedicae glnB::*Tn5*-20B* mutant (strain B12-27) that was unable to fix nitrogen. In order to clarify these contrasting results, we tested for the presence of the GlnK protein in the *glnB* or *glnK* mutant strains grown under high or low ammonium concentrations using anti-GlnK antibodies [Supplemental Figure S1]. Under the assay conditions, anti-GlnK was specific towards GlnK and did not recognize GlnB (data not shown). The GlnK protein was expressed in the wild-type strain (SmR1) grown under nitrogen-limiting conditions, but was not detected in the presence of high ammonium confirming the nitrogen-dependence of GlnK synthesis. In the case of LNglnKdel* *mutant (*ΔglnK*), GlnK was not detected in any of the assayed conditions. In the LNglnB strain, GlnK was detected only in cells grown under low ammonium concentration, a pattern similar to that of the wild-type strain. On the other hand, GlnK was not detected in the B12-27 strain in any condition tested, showing a pattern similar to the LNglnKdel mutant rather than to LNglnB. This result suggests that the Nif minus phenotype observed in the B12-27 mutant was due to the absence of the GlnK protein, and suggests that a secondary recombination event may have happened in the B12-27 strain resulting in loss of GlnK, not detected by Benelli et al. [Supplemental Reference S1].

### Method

*H. seropedicae* strains were grown in NFbHP medium containing glutamate (5 mmol/L) or NH_4_Cl (20mmol/L) as nitrogen source. Cells were harvested and disrupted by sonication. Protein concentration in cell-free extracts was determined by the Bradford method using bovine serum albumin as standard. Proteins samples were loaded onto a 12% SDS-PAGE [Supplemental Reference S2] and processed for immunoblotting as described [Supplemental Reference S3], using mice-raised polyclonal anti-GlnK and the ECL chemiluminescence detection system (GE Healthcare). The luminescent bands were visualized and recorded using a Biochemi system (UVP) and analyzed using LabWorks (UVP) version 4.0.0.8.

**References**

[S1] Benelli, E. M., Souza, E. M., Funayama, S., Rigo, L. U., and Pedrosa, F. O. (1997) Evidence for two possible *glnB*-type genes in *Herbaspirillum seropedicae*. *J. Bacteriol.* 179, 4623-4626.

[S2] Laemmli, UK. (1970) Cleavage of structural proteins during the assembly of the head of bacteriophage T4. *Nature* 227, 680-685.

[S3] Burnette, W.N. (1981) “Western blotting”: Electrophoretic transfer of proteins from sodium dodecyl sulfate-polyacrylamide gels to unmodified nitrocellulose and radiographic detection with antibody and radioiodinated protein A. *Anal. Biochem.* 112, 195-203.


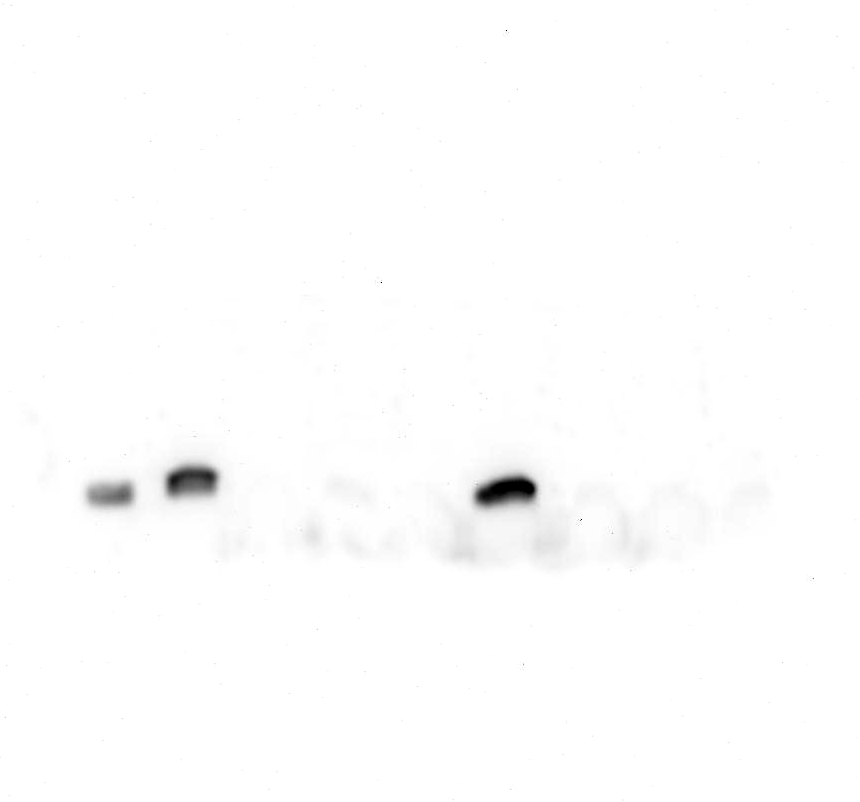


**- +**

**- +**

**- +**

**- +**

**SmR1**

**B12-27**

**LNglnB**

**LNglnKdel**

*anti-GlnK*

**Figure S1 – Immunodetection of the GlnB and GlnK proteins in *H. seropedicae* strains.** SmR1 (wild-type), LNglnB (*glnB*-Tc^R^), LNglnKdel (Δ*glnK*) and B12-27 (*glnB*) cells were grown in NFbHP medium, supplemented with 5 mmol/L glutamate (-) or 20 mmol/L NH_4_Cl (+). Cell extracts were prepared as described, and 20 mg of total protein were loaded onto SDS-PAGE. Immunodetection was carried out as described using anti-GlnK antibody and an ECL-chemiluminescent detection system.
